# Clinical, functional and salivary cortisol level changes in individuals with schizophrenia undergoing cognitive behavioral therapy for psychosis in addition to their usual antipsychotic medication

**DOI:** 10.1016/j.cpnec.2025.100312

**Published:** 2025-07-12

**Authors:** Felicia Iftene, Adriana Farcas, Simon O'Brien

**Affiliations:** Providence Care Hospital, Department of Psychiatry, Queen's University, Kingston, Ontario, Canada

## Abstract

• Evening cortisol levels assessed at baseline were higher than the levels in healthy individuals
• The morning cortisol levels showed a statistically significant increase by the end of the CBTp intervention, as participants presented with very low levels initially
• Statistically significant clinical improvement was observed at the end of the 16 weekly CBTp sessions, by comparison to the control group
• Several baseline variables were identified as correlating significantly with the main outcome measures of the CBTp intervention, including the evening cortisol level.

## 1 Background

Despite its low prevalence of 1 % globally, schizophrenia was ranked the 12th most disabling disorder by the Global Burden of Disease study in 2016. Furthermore, individuals with schizophrenia are 2–3 times more likely to die early compared to the general population, being associated with an average of 14.5 potential years of life lost [1,2]. In 2019, schizophrenia cost the United States an excess economic burden of 343 billion US dollars, including direct health care costs, non-direct health care costs (e.g. homeless shelters, law enforcement -incarceration, police protection, research and training) and indirect costs (productivity loss, unemployment, caregivers cost). Despite advancements in antipsychotics development, the recovery in schizophrenia remains often partial, with a high relapse rate. There is a need to search for new treatment protocols and possible effective augmenting techniques of the current treatment options in order to improve the quality of life and the level of functioning of these understudied population groups and decrease the costs of care.

*The rationale behind our decision to study the cortisol levels in this research.* Cortisol is the most important steroid hormone that significantly affects the body's metabolism. Fluctuations of cortisol secretion are often seen in mental disorders, and the normalization of levels correlates with the patient's health [3]. This may suggest that cortisol could be a useful biological marker, helpful in determining the likelihood of the mental illness, its impending onset, and the severity of symptoms.

Among the myriad of factors discussed in the literature, inflammation stands out as an important factor in schizophrenia pathophysiology. Stress has also been strongly highlighted as an important etiological factor, given its ability to modify the hypothalamic-pituitary-adrenal (HPA) axis response [4], with an activating effect on the cytokine-secreting microglia (Perry, 2007). Even though patients with chronic schizophrenia have an autonomic arousal response and subjective self-report of stress comparable to healthy individuals, numerous studies report that they also present with attenuated cortisol levels in response to laboratory-induced psychosocial stress [5]. While the causes of schizophrenia are complex, stress could be an important contributing factor in triggering the onset of symptoms in people who already have a predisposition to the condition [6]. Elevated cortisol secretion in some patients with schizophrenia may be, in part, the trigger for illness onset and may determine the severity of symptoms and the course of illness. It is well known that excessive cortisol levels can suppress neurogenesis, inhibit synaptogenesis and result in atypical dendritic branching and axon development [6]. Known as the stress hormone, cortisol levels regulate the body's stress response, playing other essential roles, including suppressing inflammation, regulating blood pressure and blood sugar, and controlling the sleep-wake cycle.

The cortisol awakening response impacts the stress response and resilience, blood sugar management, inflammation regulation, autoimmune development and progression. Every day, we rely on the hormone cortisol to jumpstart our morning and power us through our day. The cortisol released in the brain wakes us up, and we should get a sharp rise in cortisol about 30 min after waking, a rise called the cortisol awakening response [7]. The levels should be lower at night (when the melatonin levels rise). It was suggested that elevated levels of cortisol in individuals at clinical high risk of psychosis predict the subsequent onset of psychotic disorder. The increased cortisol level could impair brain function (thought processing, memory, and emotional regulation), suppress neurogenesis, inhibit synaptogenesis, and result in atypical dendritic branching and axon development.

The influence of antidepressants and antipsychotics on cortisol levels in healthy people and those with mental disorders was described in the literature [3]. However, the cortisol levels have never been studied in people living with psychosis, undergoing CBTp.

*Research intervention protocol.* The current Ontario Therapeutic Guidelines for schizophrenia include cognitive behavioural therapy for psychosis (CBTp) associated with neuroleptic medication. Our research uses, for the first time, to our knowledge, a new intervention protocol with 4 weekly befriending sessions preceding the 16 weekly CBTp sessions (recommended by guidelines), followed by 2 CBTp booster sessions one-month post-intervention and, respectively, three months after the end of the 16-session intervention, along with their usual medication regimen.

*The project's novelty* lies in the way we combined different already-in-use and approved interventions, in the chosen variables of interest, and their correlations with the outcome measures. We aim to examine the clinical and functional improvement in people living with schizophrenia undergoing our intervention protocol associated with their usual antipsychotic medication and to assess the changes in diurnal salivary cortisol levels.

## 2 Objectives

In this research, our primary objective was to assess clinical changes, targeting complex symptomatology (PANSS), cognitive (Cognitive Flexibility Scale, CFS) and negative symptoms (NSA-16), in participants living with schizophrenia, undergoing our intervention protocol (Cognitive Behavioral Therapy for Psychosis, CBTp X 16 weekly sessions, preceded by Befriending X 4 weekly sessions and followed by CBTp booster sessions X 2). The secondary objectives were: to assess changes in the quality of life and level of functioning before, during, and after treatment; to examine the potential changes in cortisol levels in response to treatment; to make possible correlations of the baseline data collected with the therapeutic improvement.

## 3 METHODES

This is a pilot, prospective, randomized, repeated-measures study design involving no risk and using the current guidelines-accepted therapies.

**Study population.** Individuals with a diagnosis of Schizophrenia (DSM-5 criteria) stratified by age and gender, treated with their usual medication regimen, unchanged for at least 3 months before enrolment in this study, were recruited at Providence Care, Mental Health Services, in Kingston (PCH-MHS). We expected 150 potentially eligible patients from PCH-MHS. Of the 50 patients who consented to participate, 40 were eligible, and of them, 32 were able to continue their involvement with this research, completing it.

Healthy participants (n = 32) with no psychiatric or unstable medical diagnosis were matched to the patient group by gender and age distribution and were used for lab standardization; the healthy participants were not involved in research activities (aside from saliva collection) and were not considered control group.

**Biological specimen collection.** To address the diurnal variation of cortisol, samples were collected in the morning (at 8 a.m.) and in the evening (at 10 p.m.) for the first visit and only in the morning of each subsequent visit via the SalivaBio Oral Swab method, which is a better alternative to the passive drool method because of its ease of use. This method is also proven to have the highest correlation to passive drool, and it has been validated for multiple salivary biomarkers. The complete saliva collection kit includes the oral swab, swab storage tube and sample collection instructions. The swab consists of a synthetic, hygienic, individually wrapped oral fluid collection device, which minimizes the possibility of environmental contaminants. The swab material provides ready-to-process samples by filtering out unwanted mucins, cells and other aggregates from oral fluid specimens. The device maintains the pH of the sample. The volume of sample recovered typically varies between 200 and 1000 μL depending on the individual's salivary flow rate, hydration, status, and mucus content. The participants were instructed to avoid eating, drinking, brushing teeth or taking oral medication for 1 h before saliva collection, not smoking at least 2 h before collection and not use alcohol 12 h before saliva collection.

Immediately after collection, samples were frozen below −80 °C until assayed in batch. Prior to sample testing, the samples were brought to room temperature, vortexed and then centrifuged at 1500×*g* for 15 min. Assays were performed using the Salimetrics Cortisol Enzyme Immunoassay Kit. The salivary cortisol ranges for adults, in our laboratory were 0.094–1.551 μg/dl in the morning and ND -0.359 μg/dl in the evening. ND = none detected; the minimal concentration of cortisol that can be distinguish from 0 is 0.007 μg/dl.

**Fig. 1:**
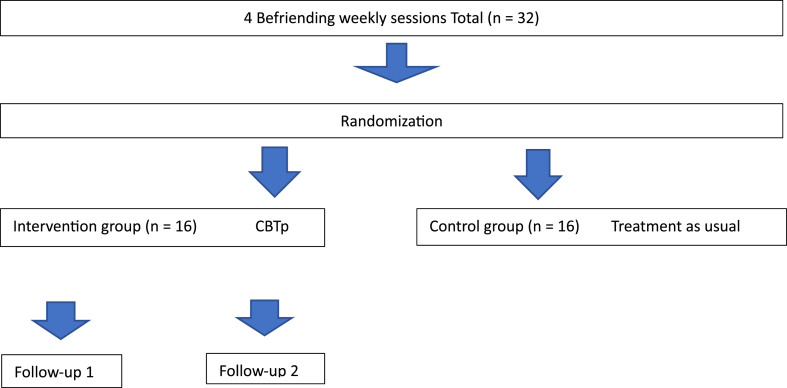
Overview of the protocol.

**Intervention protocol** (Fig. 1). After the screening visit, eligible subjects were exposed to a non-specific intervention (Befriending) for 4 weeks (one Befriending session per week). They were randomly assigned to one of two treatment arms: Control group – waitlisted for CBTp (n = 16), receiving their previous/usual treatment (no intervention, only assessments during the four designated visits) and Intervention group CBTp (n = 16). At the end, the participants from the control group were offered the option for CBTp.

The inclusions and exclusions criteria are described in Table 1.

**Table 1:** Inclusion and exclusion criteria.

Inclusion criteria	Exclusion criteria
Diagnosis of SZ and SZA (DSM V criteria)	Moderate and severe comorbid intellectual disability
Stable under current treatment for at least 3 months (no recent changes in neuroleptics)	Current suicidal ideation/plans, treatment with stimulants, metronidazole, currently undergoing hormone therapy
Age over 18–65 (participants 65+ eligible depending on performance on cognitive assessment)	Age under 18
Able to provide written informed consent, and where not possible, by proxy	Unable to provide informed consent
Ability to understand English with reading level at or above grade 6	Not fluent in English
Able to understand and comply with the requirements of the study	Undergone vagotomy or surgery on the Vagus nerve, comorbid neurological condition
Not taking benzos (hold) the morning of the intervention/investigations	History of TBI, MI, seizures, congenital heart disease, terminal/severe diseases (liver, kidney, cancer)
	Current illicit drug/substance abuse
	Current enrollment in CBTp or other formalized psychosocial intervention

Abbreviations List. *SZ: schizophrenia; SZA: schizoaffective disorder; DSM: Diagnostic and Statistical Manual of Mental Disorders; TBI: traumatic brain injury; MI: myocardial infarction; CBTp: cognitive behavioral therapy for psychosis.*

### 3.1 Experimental design, procedures and timelines

Our repeated-measures design included the following visits (V): V. 1 (screening, week 0 = W. 0); V.2 (baseline, starting befriending, W.1); V. 3 (end of befriending/starting CBTp, W.5); V.4 (CBTp 8/mid-term, W.13); V.5 (CBTp 16/end of intervention, W. 21); V. 6 (follow-up 1, W.25); V.7 (follow-up 2, W.33), as depicted in Table 2.

**Table 2:**
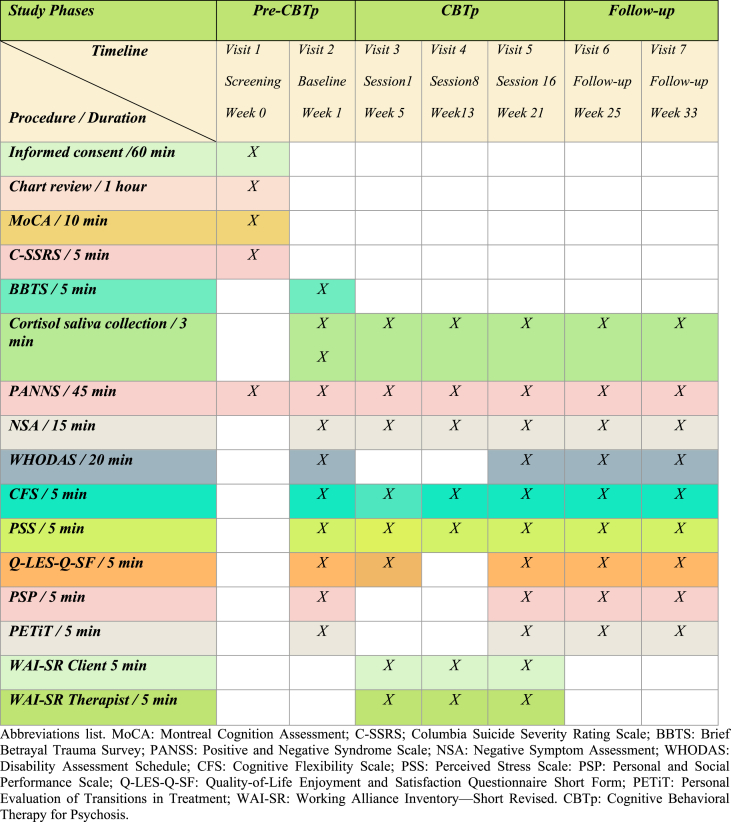
Study visits schedule, procedures and timeline.

At the Screening Visit (visit 1), eligible participants provided written informed consent for all study procedures. The chart review searched for: comorbid metabolic conditions, lab work abnormalities (HbA1c, PCR high sensitivity, cholesterol, triglycerides, HDL cholesterol), substance use, BMI, type of medication, side effects, medical and psychiatric family history.

At the Baseline Visit (Visit 2), psychosocial assessments were conducted, and the participants were randomly assigned to one of two groups: intervention or wait-list groups.

Seven visits were conducted, all of which included psychosocial measurements, but only six of them included biological specimen collection (saliva).

At the conclusion of the study, patients were discharged into standard clinical care, family physician and/or regular psychiatrist. Patients could also elect to enroll in a long-term CBTp support group.

The research included two follow-up visits (one, respectively, 3 months post-intervention).

The control/wait list group participants attended only four visits: visit 1 (screening), visit 2 (baseline), visit 3 post-befriending (pre-CBTp visit for the study group), and visit 5 (corresponding to the end of intervention visit for the study group).

The primary outcome measures were the Positive and Negative Syndrome Scale (PANSS), the Negative Symptom Assessment (NSA-16), and the Cognitive Flexibility Scale (CFS).

Secondary psychological measures: The Quality-of-Life Enjoyment and Satisfaction Questionnaire Short Form (Q-Les-Q-SF), WHO Disability Assessment Schedule 2.0 (WHODAS 2.0), Brief Betrayal Trauma Survey (BBTS), Personal and Social Performance Scale (PSP), Perceived Stress Scale (PSS) and the Personal Evaluation of Transitions in Treatment (PETiT).

Medication compliance: PETiT.

The therapeutic alliance instruments used were the Working Alliance Inventory—Short Revised (WAI-SR), both forms for client and therapist.

Screening instruments: the Montreal Cognition Assessment (MoCA) and the Columbia Suicide Severity Rating Scale (C-SSRS).

## 4 Results

**The demographics** for the 32 participants can be seen in Table 3.

**Table 3:** Demographics.

Variable	Intervention		Control	
	Number	%	Number	%
Gender				
Male	11	68.8 %	11	68.8 %
Female	5	31.5 %	5	31.5 %
Race				
White	16	100 %	14	87.5 %
Indigenous			1	6.3 %
East Asian			1	6.3 %
Education				
< High school	3	18.8 %	7	43.8 %
High school	2	12.5 %	6	37.5 %
College	6	37.5 %	1	6.3 %
University	5	31.3 %	2	12.5 %
Age				
18-30	1	6.3 %	1	6.3 %
31-55	8	50 %	6	37.5 %
>55	7	43.8 %	9	56.3 %
Housing				
Independent	11	68.8 %	10	62.5 %
With a roommate	1	6.3 %		
With family	3	18.8 %	2	12.5 %
Supported housing	1	6.3 %	4	25.0 %
Employment				
Supported employment	4	25.0 %	3	18.8 %
Volunteer	1	6.3 %	1	6.3 %
Part-time employment	2	12.5 %	2	12.5 %
No employment	9	56.3 %	10	62.5 %
Employment history				
No	3	18.8 %	4	25.0 %
Yes	13	81.3 %	12	75.0 %
Financial				
ODSP	5	31.3 %	15	93.8 %
ODSP/CPP	3	18.8 %		
ODSP/Employment income	6	37.5 %		
CPP/Old age pension	2	12.5 %	1	6.3 %
Family support				
No	4	25.0 %	6	37.5 %
Yes	12	75.0 %	10	62.5 %
Partner				
No	12	25.0 %	14	87.5 %
Yes	4	75.0 %	2	12.5 %
Friends				
No	7	43.8 %	6	37.5 %
Few	7	43.8 %	7	43.8 %
Many	2	12.5 %	3	18.8 %

The psychiatric and medical diagnoses (including BMI – weight status/obesity and lab work), psychiatric history (age of onset, number of hospitalizations), personal history of trauma and use of substances, as well as family history, are represented in Table 4. Nine individuals in each group had a BMI greater than 30; 8 individuals in the intervention group and 5 in the control group had an HbA1c greater than 6.

**Table 4:** Psychiatric and medical conditions.

Variable	Intervention		Control	
	Number	%	Number	%
Main psychiatric diagnosis				
Schizophrenia	8	50 %	10	62.5 %
Schizoaffective Disorder	8	50 %	6	37.5 %
Age at onset				
<25 years old	10	62.5 %	8	50.0 %
26–40 years old	6	37.5 %	8	50.0 %
Number of hospitalizations				
<5 times	9	56.3 %	9	56.3 %
6–10 times	7	43.8 %	4	25.0 %
>10 times			3	18.8 %
Trauma	7	43.8 %	6	37.5 %
Drug use history				
Alcohol	5	31.3 %	1	6.3 %
Alcohol, cannabis, illegal drugs	4	25.0 %	6	37.5 %
Alcohol, cannabis, illegal drugs, medication	1	6.3 %	1	6.3 %
None	6	37.5 %	6	37.5 %
Current drug use				
Cannabis, Nicotine	2	12.5 %	1	6.3 %
Nicotine	6	37.5 %	7	43.8 %
Medical Comorbidities				
Diabetes	4	25.0 %	3	18.8 %
Hypertension	4	25.0 %	3	18.8.%
Hypercholesterolemia	8	50.0 %	8	50.0 %
Psychiatric family history				
Schizophrenia/Schizoaffective Disorder	8	50.0 %	8	50.0 %
Affective Disorders	1	6.3 %	2	12.5 %
Substance use	2	12.5 %	9	56.3 %
Suicide	4	25.5 %	4	25.0 %
BMI				
Overweight (25–30)	4	25.0 %	2	12.5 %
Obese (>30)	9	56.3 %	9	56.3 %
CRP				
<1.0	4	25.0 %	4	25.00 %
>1.0	12	75 %	12	75.0 %
HbA1c				
<6.0	8	50.0 %	11	68.8 %
>6.0	8	50.0 %	5	31.2 %
Triglycerides				
<1.70	8	50.0 %	8	50.0 %
>1.70	8	50.0 %	8	50.0 %
Cholesterol				
<5.20	7	43.8 %	14	87.5 %
>5.20	9	56.3 %	2	12.5 %
HDL				
<1.20	12	75 %	12	75.0 %

The CBTp was an add-on intervention to the usual medication regimen, presented in Table 5.

**Table 5:** Current medication regimen.

Variable	Intervention		Control	
Current Antipsychotic Medication				
Clozapine	2	12.5 %	7	43.8 %
Quetiapine	3	18.8 %	1	6.3 %
Olanzapine	2	12.5 %	3	18.8 %
Aripiprazole			2	12.5 %
Paliperidone	3	18.8 %		
Asenapine	1	6.3 %		
Brexpiprazole			1	6.3 %
Risperidone			1	6.3 %
Combination of antipsychotics				
Olanzapine + Aripiprazole	1	6.3 %	1	6.3 %
Olanzapine + Chlorpromazine	1			
Quetiapine + Aripiprazole	1	6.3 %		
Quetiapine + Lurasidone + Chlorpromazine	1	6.3 %		
Aripiprazole + Paliperidone	1	6.3 %		
Long-acting antipsychotics	6	37.5 %	3	18.8 %
Antidepressive medication	5	31.3 %	3	18.8 %
Mood stabilizer	2	12.5 %		

All participants were under treatment with atypical antipsychotics, 2/3 on a monotherapy regimen, and 1/3 a combination of antipsychotics; 1/3 received a long-acting form, also 5 had an antidepressive drug and two a mood stabilizer added.

We found no clinical improvement as a result of the **befriending intervention** in our study (Table 5), as assessed by the clinical scales, with the exception of the PANSS General Anxiety dimension (we found a statistically significant decrease in the PANSS General Anxiety dimension, from pre-to post-befriending, Z = 2.12, p = 0.034, as seen in Table 6). A statistically significant reduction in anxiety appears to sustain the statistically significant improvement in quality of life observed (*Z* = 4.37, *p* = 0.001).

**Table 6:** Befriending outcome measures.

Outcome measures	Baseline		Post-Befriending		Wilcoxon	
	Median	Mean	Median	Mean	z	p
Q-LES-Q-SF	37.00	37.78	41.50	41.65	4.37	0.001
Mood	2.50	2.50	3.00	2.87	2.81	0.005
Social Relationships	2.00	1.97	2.00	2.47	3.55	0.001
Family Relations	2.00	1.87	2.00	2.63	3.51	0.001
Ability to function in daily life	3.00	2.59	3.00	3.03	2.88	0.004
Leisure time activities	2.00	1.88	3.00	2.66	3.80	0.001
Overall Sense of Wellbeing	3.00	2.53	3.00	2.94	2.70	0.007
CFS	35.00	35.00	35.00	35.00	0.059	0.953
PANSS Total	92.00	93.56	89.50	90.84	1.48	0.138
PANSS General	47.00	48.34	45.00	46.59	1.65	0.099
PANSS General Anxiety	4.00	3.78	4.00	3.59	2.12	0.034
NSA-16 Global Score	4.00	3.88	4.00	3.75	1.63	0.102
PSS	25.00	25.59	23.00	24.31	1.58	0.113

Post-befriending, the participants were randomly assigned to one of the two groups (intervention CBTp and waitlist). We conducted a data analysis to understand if there were statistically significant differences between the two groups, which may impair the validity of the post CBTp results/comparison. We assessed pre-treatment group differences by performing the Wilcoxon-Mann-Whitney test, and we found no statistically significant difference between the two groups on the rating scores for PANSS Total, morning cortisol levels, and evening cortisol levels (see means and standard deviations in Table 7).

**Table 7:** Means and standard deviation group comparison.

Outcome measure	Intervention group		Control group	
	Mean	Std. Dev	Mean	Std. Dev
***PANSS Positive***	19.63	3.69	19.25	3.00
***PANSS Negative***	24.00	4.37	25.63	3.38
***PANSS General***	47.94	8.24	45.25	6.41
***PANSS Total***	91.56	14.29	90.13	11.00
***NSA-4***	3.75	0.58	3.75	0.45
***CFS***	36.00	9.45	34.00	2.85
***Q-LES-Q-SF***	41.94	7.13	41.38	3.05
***PSS***	22.07	4.43	26.56	5.64
***Morning cortisol***	0.22	0.15	0.17	0.12
***Evening cortisol***	0.38	0.38	0.31	0.38

Note: the cortisol levels are measured in μg/dl.

**Between-groups analysis**. Scores on variables assessed in both groups (intervention and control) **before and at the end of the CBTp** intervention were analyzed using a 2x2 mixed ANOVA with the within-subject factor of scores at pre- and post-CBTp visits and the between-subjects’ factor of group (intervention or control).

As Maulchly's test indicated failed assumption of sphericity, the Greenhouse-Geisser correction was employed. Statistically significant main effects for the repeated measures and interaction between the scores at the two visits and the group factor were found for the PANSS, CFS, NSA, Q-LES-Q-SF, WHODAS 2.0, PETiT and morning cortisol, with values shown in Table 8. Means and standard deviations for these assessments can be seen in Table 9.

**Table 8:** Between-groups 2x2 mixed ANOVA results.

	Variable ∗ Visits Main Effects			Variable ∗ Group Interaction		
	*F (1, 30)*	*P*	*η*_*p*_^*2*^	*F (1,30)*	*p*	*η*_*p*_^*2*^
*PANSS Total*	56.88	<0.001	0.65	58.91	<0.001	0.66
*PANSS Positive*	33.43	<0.001	0.53	44.74	<0.001	0.60
*PANSS Negative*	22.43	<0.001	0.43	22.43	<0.001	0.43
*PANSS General*	40.32	<0.001	0.57	37.76	<0.001	0.56
*NSA*	1.79	0.191	0.05	12.76	0.001	0.30
*CFS*	39.53	0.001	0.57	13.191	0.001	0.30
*Q-LES-Q-SF*	7.99	0.008	0.21	60.30	0.001	0.66
*PETiT*	148.72	0.001	0.83	60.81	0.001	0.67
*WHODAS 2.0*	12.77	0.001	0.30	11.90	0.002	0.28
*PSP*	48.04	0.001	0.62	48.03	0.001	0.62
*Morning Cortisol*	16.12	<0.001	0.35	13.14	0.001	0.31

**Table 9:** Means and standard deviations.

*Outcome measures*	Intervention Group				Control Group			
	*Pre- CBTp*		*End of CBTp*		*Pre- CBTp*		*End of CBTp*	
	*Mean*	*SD*	*Mean*	*SD*	*Mean*	*SD*	*Mean*	*SD*
*PANSS Total*	91.56	14.29	77.25	12.10	90.13	10.99	90.25	9.75
*PANSS Positive*	19.62	3.69	16.19	2.81	19.25	3.00	19.50	3.16
*PANSS Negative*	24.00	4.37	20.75	4.63	25.62	3.38	25.63	3.03
*PANSS General*	47.94	8.24	40.31	6.33	45.25	6.41	45.13	5.67
*NSA*	3.75	0.58	3.06	0.85	3.75	0.45	4.06	0.25
*Morning Cortisol (CLM)*	0.22	0.15	0.48	0.27	0.17	0.12	0.19	0.14
*Q-LES-Q SF*	41.94	7.13	50.25	8.39	41.38	3.05	37.50	3.08
*PETiT*	24.25	5.16	42.44	5.30	25.75	3.84	29.75	5.73
*PSP*	45.00	7.98	58.38	13.22	44.38	5.98	44.38	5.98
*CFS*	35.88	3.83	43.81	2.37	34.00	2.85	36.13	3.84
*WHODAS 2.0*	66.31	15.61	52.10	15.61	59.44	8.97	59.19	11.13

**Factors correlating with therapeutic improvement**. Based on the correlation matrix analysis that included baseline and end-of-treatment variables, several significant correlations stood out with potential predictive values for the primary outcome measures in further, larger sample size studies. Due to the small sample size and data type, the Spearman's correlation test was used, as it is less prone to the influence of outliers or strange distributions.

Baseline variables correlating with PANSS scores post-intervention (Table 10). Several baseline variables were found to correlate in a statistically significant way with PANSS scores at the end of the intervention: CFS, Education, CRP, Evening Cortisol, Cholesterol, BMI with the PANSS Total scores; Education and BMI with the PANSS Positive scale scores; number of hospitalizations, CFS, Friends, Evening Cortisol, Cholesterol and CRP with the PANSS Negative scale scores; CFS, Education, Evening Cortisol, Cholesterol, BMI and CRP with the PANSS General scores. Values of Spearman's rho and significance can be seen in Table 10.

**Table 10:** Correlations between baseline variables and the PANSS scale scores at the end of the intervention.

Baseline Variable	Visit 5 PANSS Scale Correlation	ρ(14)	Sig.(2-tailed)
*Cognitive Flexibility Scale*	*PANSS Total*	*−.706*	*.002*
*Education*	*PANSS Total*	*.602*	*.014*
*CRP*	*PANSS Total*	*.591*	*.016*
*Evening Cortisol*	*PANSS Total*	*.746*	*.001*
*Cholesterol*	*PANSS Total*	*.576*	*.020*
*BMI*	*PANSS Total*	*.601*	*.014*
*Education*	*PANSS Positive*	*.605*	*.013*
*BMI*	*PANSS Positive*	*.593*	*.015*
*# Hospitalizations*	*PANSS Negative*	*.504*	*.047*
*Cognitive Flexibility Scale*	*PANSS Negative*	*−.623*	*.010*
*Friends*	*PANSS Negative*	*−.539*	*.031*
*Evening Cortisol*	*PANSS Negative*	*.664*	*.005*
*Cholesterol*	*PANSS Negative*	*.540*	*.031*
*CRP*	*PANSS Negative*	*.642*	*.007*
*Cognitive Flexibility Scale*	*PANSS General*	*−.706*	*.002*
*Education*	*PANSS General*	*.535*	*.033*
*Evening Cortisol*	*PANSS General*	*.763*	*.001*
*Cholesterol*	*PANSS General*	*.580*	*.018*
*BMI*	*PANSS General*	*.593*	*.015*
*CRP*	*PANSS General*	*.566*	*.022*

Several baseline variables were found to correlate significantly with the NSA scores at visit 5: CFS, education, number of hospitalizations, evening cortisol, cholesterol, friends, and CRP. Table 11 displays the values of Spearman's rho and significance. Variables were found to correlate significantly with the NSA scores at visit 5: CFS, Education, number of hospitalizations, Evening Cortisol, Cholesterol, Friends, and CRP. Table 11 shows the values of Spearman's rho and significance.

**Table 11:** Correlations between baseline variables and the NSA scores at the end of the intervention.

Baseline Variable	Visit 5 NSA Scale Correlation	ρ(14)	Sig.(2-tailed)
*Cognitive Flexibility Scale*	*NSA*	*−.639*	*.008*
*Education*	*NSA*	*.583*	*.018*
*# Hospitalizations*	*NSA*	*.517*	*.040*
*Evening Cortisol*	*NSA*	*.690*	*.003*
*Cholesterol*	*NSA*	*.591*	*.016*
*Friends*	*NSA*	*−.534*	*.033*
*CRP*	*NSA*	*.738*	*.001*

Baseline variables correlating with CFS scores post-intervention. Education and Evening Cortisol levels were the two baseline variables correlating significantly with the CFS scores at visit 5. The values of Spearman's rho and significance are seen in Table 12.

**Table 12:** Correlations between baseline variables and the CFS scores at the end of the intervention.

Baseline Variable	Visit 5 CFS Correlation	ρ(14)	Sig.(2-tailed)
*Education*	*CFS*	*−.544*	*.030*
*Evening Cortisol*	*CFS*	*−.510*	*.044*

Other significant correlations (Table 13). Morning cortisol levels at the end of the intervention correlated negatively with the number of hospitalizations, CRP levels and PANSS Negative scores at baseline and positively with having friends.

**Table 13:** Correlations between baseline variables and Morning Cortisol levels at the end of the intervention.

Baseline Variable	Visit 5 Morning Cortisol Correlation	ρ(14)	Sig.(2-tailed)
*# Hospitalizations*	*Morning Cortisol*	*−.515*	*.041*
*Friends*	*Morning Cortisol*	*.623*	*.010*
*CRP*	*Morning Cortisol*	*−.593*	*.015*
*PANSS Negative*	*Morning Cortisol*	*−.652*	*.006*

**The predictive value of the correlating variables was further explored through simple linear regression**. However, most of them did not reach statistically significant values due to the small sample size and influential outliers. Notable exceptions were the following baseline variables: Education, # Hospitalizations, and BMI.

***Education*** level was found to have an impact on the PANSS Positive scores at visit 5, with.

*F* (1,15) = 5.35, *p* = 0.037, (*t* = 7.17*, p* < 0.001), the adjusted R^2^ indicating that 22.5 % of variance in PANSS Positive scores can be explained by the variance in the level of education, with the regression equation: PANSS Positive scores at visit 5 = 12.44 + (1.3 x education level).

For the NSA scores at visit 5, education was also found to have predictive value, with *F*(1,15) = 6.97, *p* = 0.19, *(t* = 3.58, *p* = 0.03), the adjusted R^2^ indicating that 28.5 % of variance in NSA scores can be explained by the variance in the level of education, with the regression equation: NSA scores at visit 5 = 1.81 + (0.44 x education level).

Education level was also found to have predictive value for the CFS scores at visit 5, with.

*F* (1,15) = 6.80, *p* = 0.021, (*t* = 33.45, *p* < 0.001). The regression model showed that 27.9 % of the variance in the CFS scores at visit 5 can be explained by the level of education at baseline, with the following regression equation: CFS scores at visit 5 = 47.25 + (−1.22 x education level), a lower education level predicting a higher CFS score.

The ***number of hospitalizations*** at baseline (# Hospitalizations) was found to have a predictor value for the PANSS Negative scale scores at visit 5, with *F* (1,15) = 4.725, *p* = 0.047, (*t* = 12.638, *p* < 0.001). The adjusted R^2^ value indicated that 19.9 % of variance in the PANSS Negative scores at visit 5 could be explained by the variance in the number of hospitalizations at baseline, with the following regression equation: PANSS Negative scores at visit. 5 = 18.5 + (4.5 x number of hospitalizations – with higher number of hospitalizations predicting higher values of PANSS Negative scores.

The ***BMI*** value at baseline was found to have predictive value for the PANSS General scores at visit 5, with *F* (1,15) = 5.56, *p* = 0.033 (*t* = 2.16, p = 0.048). The adjusted R^2^ indicates that the variance in BMI can explain 23.3 % of the variance in PANSS General scores. The regression equation for this model was: PANSS General scores at visit 5 = 19.41 + (0.69 x BMI).

As expected, the evening cortisol levels were found to correlate significantly with family support, having friends and the number of hospitalizations. Cholesterol values at baseline correlated with the number of hospitalizations, HbA1c values, having friends, and age of onset.

## 5 Discussions

The primary purpose of this project was to execute a two steps interventional model pilot study that can innovatively create, identify, and define recovery profiles for individuals with schizophrenia undergoing Befriending and CBTp, a talk therapy treatment, in addition to the current medication regimen, as a method to improve treatment approaches and treatment trajectories. An avenue not explored in the literature, but approached by our current research, is the *use of befriending as a pre-intervention tool*, preparing patients for accepted interventions like CBT. The interactions with a volunteer in the time leading to the clinical intervention would open the door to the therapeutic engagement essential for therapeutic change, set interaction routines in place and respond to feelings of social isolation [6]. In this context, adequate resources would be used for appropriate outcomes and ensure a more comprehensive and practical approach. The Befriending was conducted in our study by undergraduate students, specifically and intensively trained for 14 days to apply the protocol developed for the study.

*CBTp* is a validated, safe, efficacious, and impactful talk therapy with a strong evidence base [8]. In terms of the adoption of CBTp in Ontario, Canada, CBTp was only added to treatment guidelines in 2016 for the treatment of adult schizophrenia (*Schizophrenia Care for Adults in Hospital - Health Quality Ontario (HQO)*, n.d.).

*The therapeutic improvement*, in our study, was defined as statistically significant changes in some of our primary as well as secondary outcomes. To capture the progress in clinical improvement, we chose two validated scales that show high reliability and sensitivity to schizophrenia symptomatology – the PANSS and the NSA. The CFS was added as an additional measure of executive function. We considered participants to be good responders to the intervention if there was an increase in PANSS General Scores of 20 % or more of their baseline scores at the end of the intervention, with an improvement in scores of the PANSS Positive and PANSS Negative scales of at least 15 % compared with the baseline. Half of our participants in the intervention group met these criteria. The therapeutic improvement also included a 20 % decrease in scores for the NSA scale (with 7 participants in the intervention group meeting this criterion) and a 20 % increase in CFS scores at the end of the intervention, compared to baseline (with half of the participants in the intervention group showing this increase).

*The highest reductions in all PANSS dimensions – Total, Positive, Negative and General*-were found at the end of the CBTp intervention, consistent with the expectation of clinical improvement. Although all participants in the intervention group showed improvement, 8 of them reached the 20 % and above threshold of clinical improvement, with the highest improvement in the PANSS Positive scale (22.37 % reduction on average), followed by the PANSS General scale (average of 17.78 % reduction) and the PANSS Negative scale (average of 15.27 % reduction). Consistent with our findings, a recent study exploring the minimum clinically important change in negative symptoms in a 26-week-long, double-blind study with 454 patients with schizophrenia found a cut-off value of 15 % [9].

One of the distinguishing factors that needs to be mentioned is that of these 8 participants with high improvement (20 % and above reduction in PANSS total scores), 6 had an antidepressive medication added to their antipsychotic pharmacological regimen; they also had a late onset of their disorder (age between 26 and 40 years old), and fewer number of hospitalizations compared to those who did not improve clinically in a statistically significant way. Schizoaffective disorder was the predominant diagnosis (6 of the 8 participants who significantly improved, compared to 2 of the remaining 8). The gains, still statistically significant at the two follow-ups (for all 16 participants undergoing CBTp), showed an upward tendency, implying that the clinical therapeutic improvements achieved at the end of the intervention will gradually fade away.

Possible predicting factors emerged during our statistical analysis (CFS, Evening cortisol, education, cholesterol, number of hospitalizations, BMI at baseline), related to the PANSS total decreased score, reflecting the expected clinical improvement. Evening cortisol levels at baseline (see values in Table 10) correlated positively with the PANSS Total scores post-intervention, showing a possible relation between an abnormal diurnal cortisol variation and more severe clinical presentation or lack of clinical improvement. Abnormal diurnal cortisol variations have been shown to accompany psychiatric disorders, including schizophrenia, and normalization of its levels correlates with clinical improvement [3].

High cholesterol levels as well as BMI at baseline correlated positively with the PANSS Total scores post-intervention, showing the significant impact of the metabolic state on clinical response to therapy. Moreover, lastly, the education level was also found to correlate positively with the PANSS Total scores at the end of the intervention, consistent with literature suggesting difficulties in positive symptoms remission in individuals with a higher education level, as they tend to present with stronger defence mechanisms [10]. However, a predictor value for these variables could not be reached due to the small sample size of participants (we may need at least 30 participants undergoing the intervention).

*The Cognitive Flexibility Scale.* CFS scores at baseline correlated negatively with the PANSS Total scores at the end of the intervention (higher initial CFS scores with decreased PANSS Total scores post-intervention). A core cognitive control function, supported by the brain networks of the whole brain, cognitive flexibility has been shown to be deficient in individuals with schizophrenia, with distinct functional connectivity patterns compared to healthy individuals [11]. Lower brain global connectivity was also found to correlate with clinical symptoms and severity [12]. Based on the current understanding of the origin of cognitive deficits in schizophrenia, cognitive flexibility becomes an important reflection of the impaired communication between distinct brain regions, leading to an inability to connect separate psychological and neurobiological constructs into a cohesive whole, necessary for daily functioning [13]. Improvements in cognitive flexibility scores as a result of learning-like experiences, as is the case with a CBT intervention, may imply neural plastic changes that would need consistent, systematic efforts to solidify, long after the end of the intervention. Our findings also highlight the importance of establishing a screening threshold value in scores on the CFS, supporting improvements on clinical scales in individuals undergoing a CBTp intervention. Numerous studies show that individuals with this diagnosis perform worse on many standardized neuropsychological assessments compared to healthy individuals, as well as with individuals suffering from different psychiatric disorders Wobrock et al., 2009; [14]. Defined as the ability to adapt to a changing environment cognitively, cognitive flexibility is reduced in various psychiatric disorders, including schizophrenia [15]. Shown to be involved in the successful navigation of social interactions and norms, cognitive flexibility was assessed in this study to explore its amenability to change under CBTp intervention. We found a significant improvement in cognitive flexibility scores throughout the intervention, with an expected plateau at the two follow-ups (significant correlations were found between CFS scores at the end of intervention, and the evening cortisol level (a level lower than 0.36 μg/dl), cholesterol level (lower than 5.0 mmol/l) and education level (high school and college level) of our participants at baseline. Keeping in mind that the cortisol levels in our sample were found to be reversed (lower levels in the morning and higher in the evening), the connection between a more functional level of cortisol, education and cognitive functions becomes evident.

*The Negative Symptoms Assessment Scale.* As one of the main distinguishing categories of symptoms in schizophrenia is the negative symptomatology, the NSA-16 scale was used in our study, considering its sensitivity to the changes in negative symptoms over time. Rated to be one of the most effective rating scales to measure negative symptoms, NSA is the only instrument with a single item measuring the global or overall severity of negative symptoms [16], which was used in our study to highlight changes over time. Studies in patients with schizophrenia have shown a clear association between negative symptoms and poor social functioning, increased likelihood of hospital admission, and readmission, as well as longer duration of admission (Aleman et al., 2016). The importance of identifying effective therapeutic approaches to address negative symptoms in psychotic disorders is consistently highlighted in the literature [17]. Aligning to this imperative, our aim in this study was to explore if a 16-weekly, individually tailored CBTp session would significantly impact the negative symptoms, as assessed with the NSA instrument. Our findings showed a gradual yet significant reduction in NSA scores in the intervention group, throughout the study, with the highest improvement at the end of the intervention and a large effect size.

Possibly predicting factors for NSA (to be explored in larger studies), several baseline variables correlated significantly with decreased NSA scores at the end of the intervention: evening cortisol, cholesterol levels, having friends, number of hospitalizations, education and CFS. A lower level of evening cortisol (under 0.36 μg/dl), lower cholesterol (under 5.0 mmol/l), having more than one friend, less then 5 hospitalizations, and a higher cognitive flexibility score at baseline (between 25 and 44) showed a statistically significant correlation with improvement in NSA scores at the end of the intervention.

Aside from the main clinical outcomes measured by PANSS, NSA-16 and CFS, additional information was explored by assessing trauma experiences with BTTS and perceived stress with the PSS, but no statistically significant correlations could be established. However, the PSS scores showed the lowest values at the end of the intervention, only to show an increasing slope again at the follow-up visits.

*Quality of Life and Functional Improvement*. Quality of life, as assessed by the Q-LES-Q-SF, was found to significantly improve by the end of the CBTp intervention in our study, with the highest scores at follow-up, showing good retention of improvement well past the end of the intervention, similar to findings in other studies (Freeman et al., 2015; [[18], [19], [20]]; Agbor et al., 2022). Although the efficacy of a CBT approach in psychosis has been consistently explored, since its first use in a psychiatric population, not all studies look explicitly into quality-of-life changes, as the focus is usually placed on markers of clinical improvement [[21], [22]]. Consistent with research reports supporting the amenability of CBTp in schizophrenia, our findings show a consistent improvement in quality of life as reported by our participants in the intervention group. The items of the Q-LES-Q-SF showing the most significant improvement in our study were related to managing medication, activities of daily living, family relationships, falling, and life satisfaction (with an improvement of more than 20 %) post-intervention.

The level of functioning in the daily life activities, as assessed by the PETiT scale, the PSP scale, as well as the WHODAS 2.0, also showed a significant improvement at the end of the intervention, further highlighting a positive impact the sessions had on the everyday life experiences of the participants in the study. An important piece of this puzzle, provided by the third-party information collected through the PSP, completed the subjective picture offered by the self-report scales (Q-LES-Q-SF and WHODAS 2.0) with a clinician perspective. The most important domain showing improvement in our study was the domain of personal and social relationships, going from a “manifest difficulty (but not marked)” at baseline to “mild” at the end of the intervention (20 % reduction). Our results align with the highly relevant goal of recovery of social/community functioning in individuals with schizophrenia, as a substrate for improved quality of life and reduced costs for the healthcare system, as highlighted in the literature ([23]; Wijnen et al., 2018 [[24], [25]]).

*Salivary cortisol*. Research seems to have reached consensus about the HPA axis dysfunction in schizophrenia [6], numerous reports showing that individuals with chronic schizophrenia present with attenuated cortisol levels in response to lab-induced psychosocial stressors, despite an autonomic arousal response and subjective self-report of stress comparable to healthy individuals [26]. Similar to these reports, our findings showed a reversed pattern of cortisol levels at baseline, by comparison with healthy individuals, with lower levels of cortisol in the morning and higher levels in the evening (morning cortisol pre-CBTp M = 0.22 μg/dl, and evening cortisol with M = 0.38 μg/dl. As the CBTp intervention progressed, the morning cortisol levels increased, peaking at the end of the intervention (M = 0.48 μg/dl, with statistically significant main effects between the pre- and post-intervention visits (*F(1,30*) = 16.12, *p* < 0.001) and statistically significant interaction between visits and intervention group (*F(1,30)* = 13.14, *p* = 0.001). Involved in a myriad of physiological interactions, with its powerful anti-inflammatory and metabolic effects, cortisol has been seen as a key determinant of health [[27], [28]].

A few baseline variables were also found to reach statistical significance level as correlating negatively with the morning cortisol levels at the end of the intervention: having friends, lower CRP and cholesterol levels as well as PANSS Negative scores. Evening cortisol levels were found to correlate significantly with the level of family support, having friends and the number of hospitalizations. These findings show a complex interplay of social and biological factors with potential impact on the cortisol levels.

*Baseline possible indicators of therapeutic improvement summary.* Considering the importance of an appropriate allocation of resources available in the healthcare system for rehabilitation of individuals with schizophrenia, this pilot study aimed at bringing some clarity and direction in the process of selecting individuals who would fit the criteria of good responders to an intervention like CBTp.

**Fig. 2:**
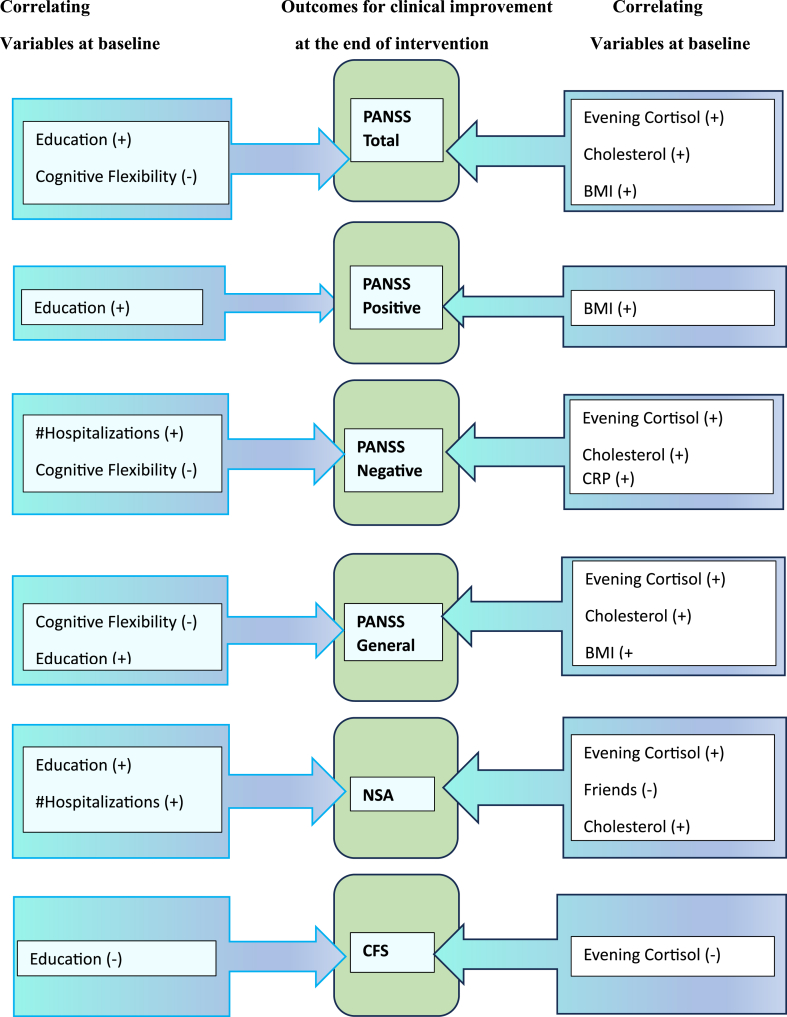
Variables correlating with therapeutic improvement.

Based on the results we obtained in this study, it was important for us to highlight factors distinguishing participants in the intervention group who seemed to respond well to the CBTp strategies employed over the 16 weekly sessions. Beyond the predictive factors identified for this group as a whole (education, number of hospitalizations, and BMI at baseline), important correlating factors can be seen in Fig. 2.

A higher cognitive flexibility score at baseline, along with lower evening cortisol, cholesterol, and CRP levels, lower BMI and, unexpectedly, education (lower than University level), was associated with a significant decrease in the PANSS Total scores at the end of the intervention. The BMI and education were also significantly correlated with the PANSS Positive and General scales. A lower number of hospitalizations, lower evening cortisol, cholesterol and CRP levels, a higher cognitive flexibility score at baseline as well as having supportive friends correlated significantly with the decreased PANSS Negative scale and the NSA scores at the end of the intervention, suggesting that a differentiation between individuals who will see a reduction in negative symptoms and those who will not, may be possible. However, future larger studies are needed to clearly depict variables like these as predictors.

### 5.1 Limitations

One of the main limitations of this study, not unlike numerous other studies in psychiatry research, is the small sample size, thus limiting the statistical power and effects of changes observed. Although numerous baseline variables indicated potential predictor values, statistical significance could not be reached due to the stringency of non-parametric testing of samples under 30 participants. Another limitation is due to the time when the research activities were carried out, it happened during the COVID pandemic when we had to adjust protocols, deal with a significant crisis in the laboratory, lose some of the important partners and increase the costs.

### 5.2 Further directions

Replicating this research protocol with a larger sample (N = 30) would allow for greater specificity and rigour in distinguishing predictors, suggesting clear selection criteria for CBTp candidates.

## 6 Conclusions

Without being this research's primary focus, befriending was identified as providing an opportunity for increased social interactions and the development of healthy social relationships, suggesting that it may be considered a complementary or supplementary intervention for patients with schizophrenia, especially when CBT is not readily available. A protocol involving befriending as a pre-CBTp intervention tool was suggested, as a preparatory stage addressing social, interactional skills necessary for the more involved therapeutic engagement of the CBTp.

Statistically significant clinical improvement was observed at the end of the 16 weekly CBTp sessions compared to the control group, as assessed with the PANSS, NSA, and CFS scales. The morning cortisol levels showed a statistically significant increase by the end of the CBTp intervention, as participants presented with very low levels initially, consistent with schizophrenia presentation documented in the literature.

Evening cortisol levels assessed at baseline also presented as higher than the levels in healthy individuals, a testament to the abnormal diurnal variation of cortisol in individuals with schizophrenia and the involvement of the HPA axis in the pathophysiology of this disorder.

Several baseline variables were identified as correlating significantly with the primary outcome measures of the CBTp intervention: evening cortisol level, cholesterol, BMI, number of hospitalizations, having friends, cognitive flexibility, and education.

## CRediT authorship contribution statement

**Felicia Iftene:** Visualization, Resources, Funding acquisition, Writing – original draft, Software, Investigation, Conceptualization, Validation, Project administration, Formal analysis, Writing – review & editing, Supervision, Methodology, Data curation. **Adriana Farcas:** Writing – original draft, Project administration, Data curation, Writing – review & editing, Software, Formal analysis, Visualization, Methodology, Conceptualization, Validation, Investigation. **Simon O'Brien:** Software, Funding acquisition, Supervision, Investigation, Writing – original draft, Resources, Data curation, Validation, Project administration, Conceptualization.

## Ethics statement

This research was reviewed by Queen's University 10.13039/100018491Health Sciences and Affiliated Teaching Hospitals Research Ethics Board (HSREB), as a part of the Research project 369826, 2019 10.13039/501100000109PCC Rsh Innovation Grant, TRAQ DSS #6030572 titled “Innovative Pathways to Impactful Treatment Of Chronic Schizophrenia: Disrupting The Status-Quo Moving Toward Biologically-Driven, Combined Pharmacological And Non-Pharmacological Therapeutic Approaches To Define Markers Of Therapeutic Improvement In Cognitive Behavioral Therapy For Psychosis Promoted Recovery”.

## Funding

Providence Care Innovation Grant 2019.

*“Innovative pathways to impactful treatment of chronic schizophrenia: disrupting the status-quo moving toward biological-driven, combined pharmacological and non-pharmacological therapeutic approaches to define markers of therapeutic improvement in cognitive behavioral therapy for psychosis promoted recovery*” ($60000)

## Declaration of competing interest

The authors declare that they have no known competing financial interests or personal relationships that could have appeared to influence the work reported in this paper.
